# Direct lysis of 3D cell cultures for RT-qPCR gene expression quantification

**DOI:** 10.1038/s41598-023-28844-1

**Published:** 2023-01-27

**Authors:** Fien Gysens, Lisa Ostyn, Ellen Goeteyn, Eva Blondeel, Justine Nuyttens, Olivier De Wever, Eric de Bony, Aurélie Crabbé, Pieter Mestdagh

**Affiliations:** 1 OncoRNALab, Cancer Research Institute Ghent (CRIG), Ghent, Belgium; 2 Department of Biomolecular Medicine, Ghent University, Ghent, Belgium; 3 Laboratory of Pharmaceutical Microbiology, Department of Pharmaceutical Analysis, Ghent University, Ghent, Belgium; 4 Laboratory for Experimental Cancer Research, Department of Human Structure and Repair, Ghent University, Ghent, Belgium

**Keywords:** RNA, Cell biology

## Abstract

In vitro cell culture experiments are widely used to study cellular behavior in most biological research fields. Except for suspension cells, most human cell types are cultured as adherent monolayers on a plastic surface. While technically convenient, monolayer cultures can suffer from limitations in terms of physiological relevance, as their resemblance to complex in vivo tissue structures is limited. To address these limitations, three-dimensional (3D) cell culture systems have gained increased interest as they mimic key structural and functional properties of their in vivo tissue counterparts. Nevertheless, protocols established on monolayer cell cultures may require adjustments if they are to be applied to 3D cell cultures. As gene expression quantification is an essential part of many in vitro experiments, we evaluated and optimized a direct cell lysis, reverse transcription and qPCR protocol applicable for 3D cell cultures. The newly developed protocol wherein gene expression is determined directly from crude cell lysates showed improved cell lysis compared to the standard protocol, accurate gene expression quantification, hereby avoiding time-consuming cell harvesting and RNA extraction.

## Introduction

Monolayer cell cultures, conventionally obtained by growing cells on a plastic two-dimensional (2D) surface, are widely used in a broad range of in vitro experiments to study disease onset, progression and treatment, but their resemblance to the in vivo morphology and function of tissues is limited^1,2^. To overcome this limitation, three-dimensional (3D) cell culture systems are increasingly used, which have a greater resemblance to the in vivo parental tissue that they mimic in terms of gene expression, signaling, metabolism and cellular topology^3^. Various 3D cell culture systems have been developed to generate physiologically relevant cell culture models^4^. Three-dimensional cell cultures are frequently based on the formation of cellular aggregates, where cells are cultured in the presence or absence of a scaffold-hereby promoting their differentiation. One such system to generate 3D cellular aggregates is the rotating wall vessel (RWV), which is an optimized suspension culture wherein cells are typically cultured on porous collagen-coated microcarrier beads^5–7^. A wide range of tissues have been mimicked using RWV-based 3D cell cultures, using either cell lines or primary cells. This includes alveolar and bronchial tissues^8,9^, small and large intestine^10,11^, heart^12^, liver^13^, female reproductive tract^14^, prostate^7^ and bone^15^. RWV-derived models were found to reflect the in vivo human tissues and organs more closely than their 2D monolayer counterparts, as demonstrated through increased apical and basolateral polarity, tissue barrier function (i.e. localized expression of tight/adherence junctions), gene expression, cytokine production, and responses to viral and bacterial pathogens/commensals, antimicrobials and microbial products (incl. cytotoxicity)^10,16–19^. Other systems to generate in vivo-like cellular aggregates include organoids or spheroids, where cells cluster in the absence of beads, typically using a hydrogel that mimics the composition of the basement membrane matrix, or using other techniques, such as hanging drops and microfluidics^20^. Regardless of differences in the methods used for the generation of 3D cell cultures, these models typically have a closer resemblance to in vivo cells, tissues and organs because of their structural organization and enhanced differentiation, and are therefore physiologically relevant for in vitro experiments^20,21^.

Gene expression quantification is an important tool to study biological processes in cellular systems, for example the response of cellular systems to external stimuli, compounds or gene perturbation. To quantify gene expression in 3D cell cultures in high-throughput settings, reverse transcription (RT) and quantitative polymerase chain reaction (qPCR) applied directly to crude lysates is more time-efficient compared to cell harvesting and RNA extraction^22^. Direct cell lysis, RT, and qPCR have previously been established to quantify gene expression in monolayer cell cultures^22–25^. To assess the performance of this method in 3D cell cultures, we evaluated and adapted an existing protocol^26^ and compared performance to a standard RNA extraction workflow. We found that direct lysis is less sensitive compared to standard RNA extraction but results in accurate gene expression measurements.

## Results

We used two different respiratory epithelial cell lines, A549 and BEAS-2B, to evaluate and optimize direct cell lysis and RT-qPCR of 3D cell cultures. A549 are alveolar type II cells originally derived from a patient with a lung adenocarcinoma, while BEAS-2B are immortalized bronchial epithelial cells, generated from a healthy individual. These cell lines were cultured in a rotating wall vessel on the surface of collagen-coated microcarrier beads to form 3D cell aggregates (Supplemental Fig. 1A). To assess the performance of direct cell lysis for RT-qPCR-based gene expression quantification in 3D cell cultures, we assessed the efficiency of DNAse treatment and compared the expression of different genes following either conventional RNA extraction (miRNeasy kit, Qiagen) or direct lysis (two versions of the SingleShot protocol, Bio-Rad^26^).

### DNA contamination

We first tested the efficiency of DNase treatment in the two lysis protocols compared to an on-column DNase treatment during RNA extraction. As demonstrated previously, DNAse treatment of crude cell lysates is essential to reduce genomic DNA contamination in subsequent RT and qPCR reactions^22^. To this end, we performed qPCR for four genes directly on cell lysate or extracted RNA without including a reverse transcription step. On-column DNAse treatment, which we considered as the gold standard, showed the best performance overall (i.e. lowest DNA signal across genes and samples), with Cq-values ranging from 35.5 cycles to not detected, depending on the gene and cell line (Supplemental Fig. 2). In A549 cells, DNAse treatment of the lysates was most efficient when applying the version 2 of the protocol and this difference was significant for all four markers. Cq-values ranged from 27.95 cycles to not detected, with an average delta Cq compared to the gold standard approach of 2.51 cycles. In BEAS-2B cells, lysis protocol version 1 resulted in the most efficient DNase treatment, but this difference was only significant for 2/4 markers. Cq-values ranged from 30.6 cycles to not detected and an average delta Cq compared to the gold standard of 3.06 cycles.

### Sensitivity

**Figure 1:**
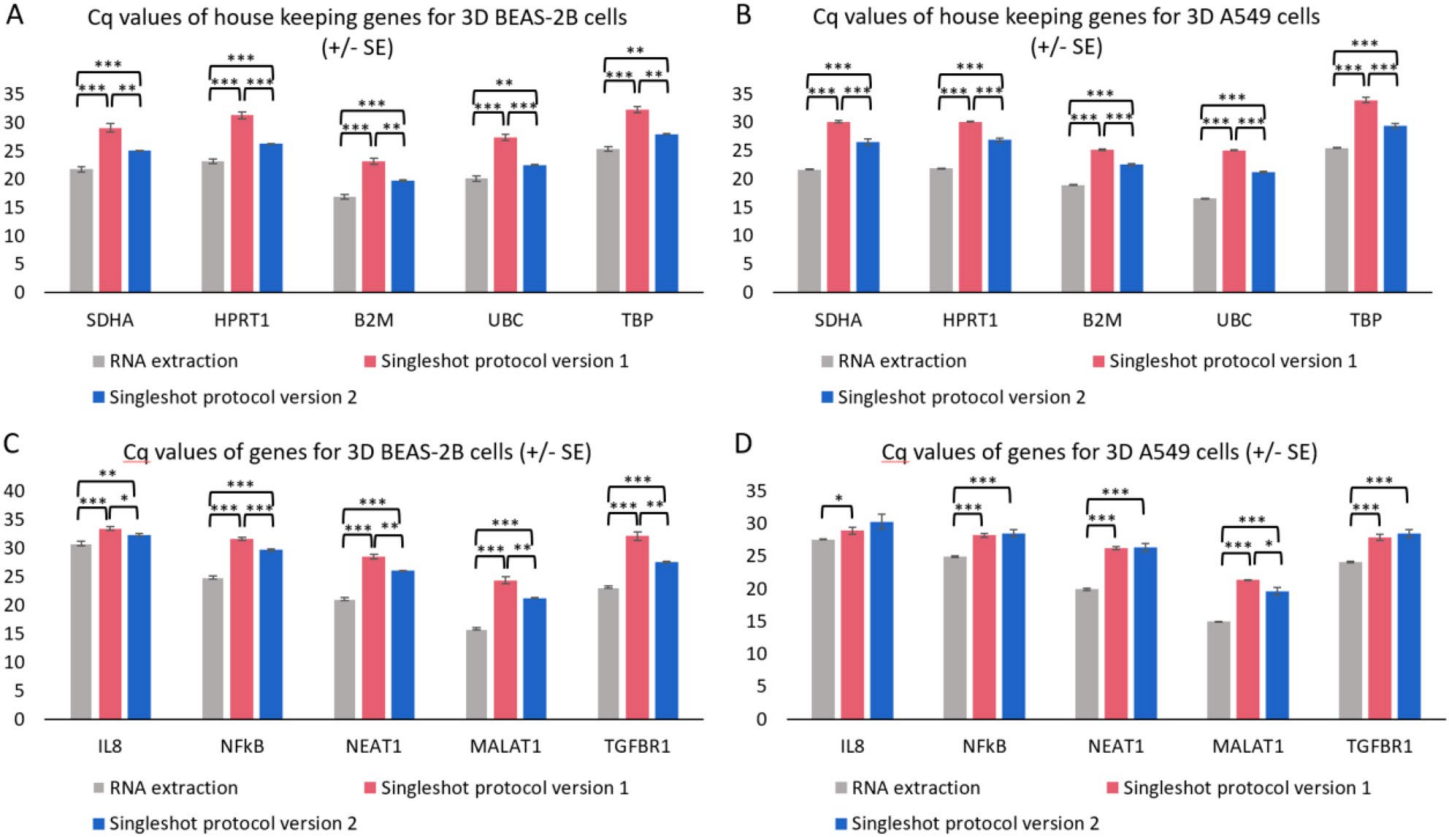
Cq values for five housekeeping genes (TBP, UBC, SDHA, HPRT1, and B2M), measured in two cell lines (BEAS-2B (**A**) and A549 (**B**)) on RNA extracted from 3D cell cultures (grey), lysates generated using the Singleshot protocol version 1 (red) or lysates generated using the Singleshot protocol version 2 (blue). Cq values for five additional genes (IL8, NFkB, NEAT1, MALAT1 and TGFBR1), measured in two cell lines (BEAS-2B (**C**) and A549 (**D**)) on RNA extracted from 3D cell cultures (grey), lysates generated using the Singleshot protocol version 1 (red) or lysates generated using the Singleshot protocol version 2 (blue). Data are presented as mean ± SE with *P < 0.05; **P < 0.01 and ***P < 0.001 (n = 3).

Second, we evaluated the efficiency of direct cell lysis on 3D cell cultures from both BEAS-2B and A549 cells. As a reference, RNA from the same 3D cell cultures was extracted using the miRNeasy kit. Following RT of crude cell lysates or extracted RNA, we quantified the expression of five housekeeping genes by qPCR, i.e. TBP, UBC, SDHA, HPRT1 and B2M (Fig. 1A,B). We observed significantly lower Cq values for all five genes for both 3D cultures on extracted RNA compared to cell lysates from the version 1 protocol (*P* < 10^−4^ paired t-test), suggesting lower sensitivity for lysis compared to extraction. The average delta Cq between lysis and extraction was 7.58 cycles, or a 191-fold reduction in sensitivity. We then adapted the lysis procedure to improve lysis efficiency by introducing a lysate mixing step (see “Materials and Methods” for details) and called this version 2. This resulted in an average decrease in Cq-values of 3.95 cycles compared to the version 1 lysis protocol, or a 15-fold increase in lysis efficiency (*P* < 10^−4^ paired t-test). The optimized lysis procedure drastically improved the sensitivity of the method and was on average 12-fold less sensitive than standard RNA extraction. We further compared these two versions of lysis by quantifying the expression of five more biologically relevant genes (IL8, NFkB, NEAT1, MALAT1 and TGFBR1). For BEAS-2B cells there still is a clear advantage in using version 2 of the protocol as demonstrated by significantly lower (*P* < 0.05) Cq values. For the A549 cell line, only one of the five genes showed a lower Cq value with version 2 (Fig. 1C,D).

### Accuracy

**Figure 2:**
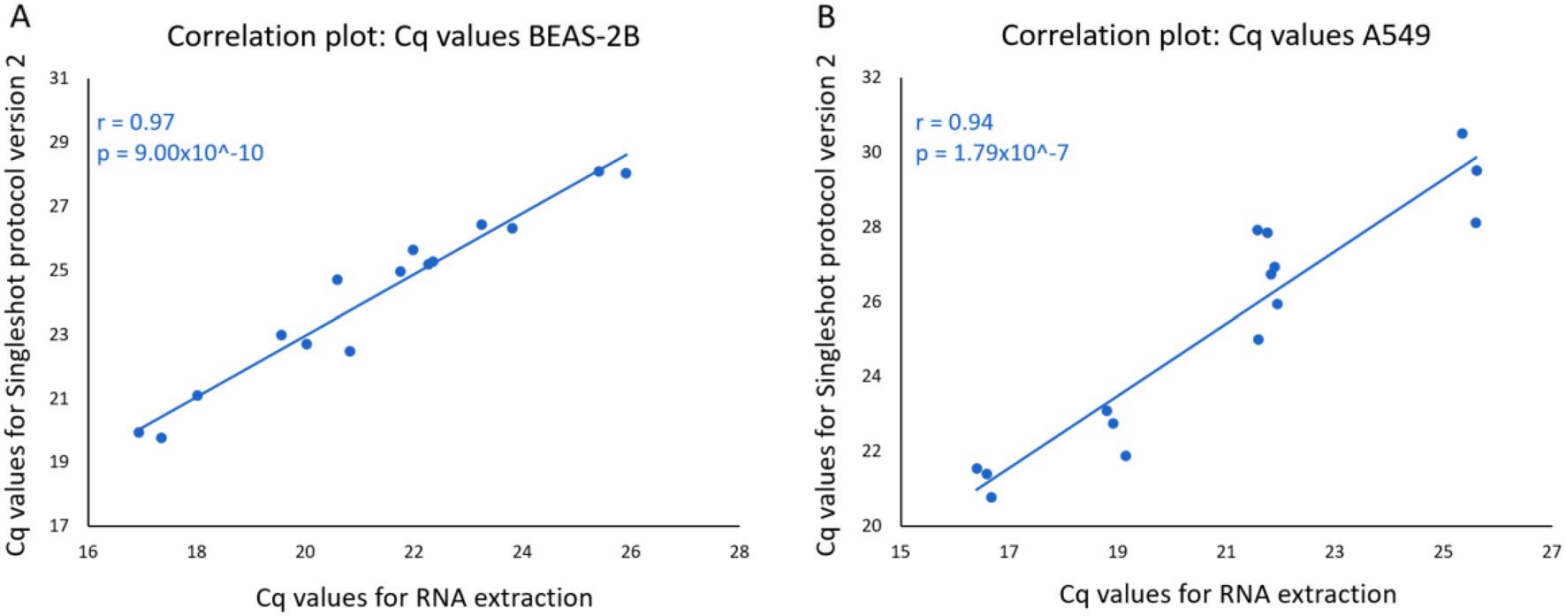
Correlation between Cq values from extracted RNA and lysate for lysis protocol version 2 in two different cell lines (BEAS-2B (**A**) and A549 (**B**)).

The ability to accurately quantify relative gene expression between 3D culture conditions starting from crude cell lysates was assessed based on expression of all five housekeeping genes in both cell lines and for each of the methods (i.e. RNA extraction with the miRNeasy kit and cell lysis with either the Singleshot protocol version 1 or version 2). Cq-values were first compared between methods in each of the 3D cell cultures (A549 and BEAS-2B cells). We observed a significant positive correlation between Cq-values obtained from extracted RNA and Cq-values obtained from direct lysis, both for the version 1 and 2 of the lysis protocol (Fig. 2 and supplemental Fig. 3). Pearson correlation coefficients for the second version (A549: R = 0.94, *P* = 1.79E−07; BEAS-2B: R = 0.97, *P* = 9.00E−10) were not significantly different than for the first version (A549: R = 0.96, *P* = 1.36E−8; BEAS-2B: R = 0.96, *P* = 2.19E−08). These results demonstrate accurate quantification of relative gene expression levels when applying direct lysis in 3D cell cultures.

**Figure 3:**
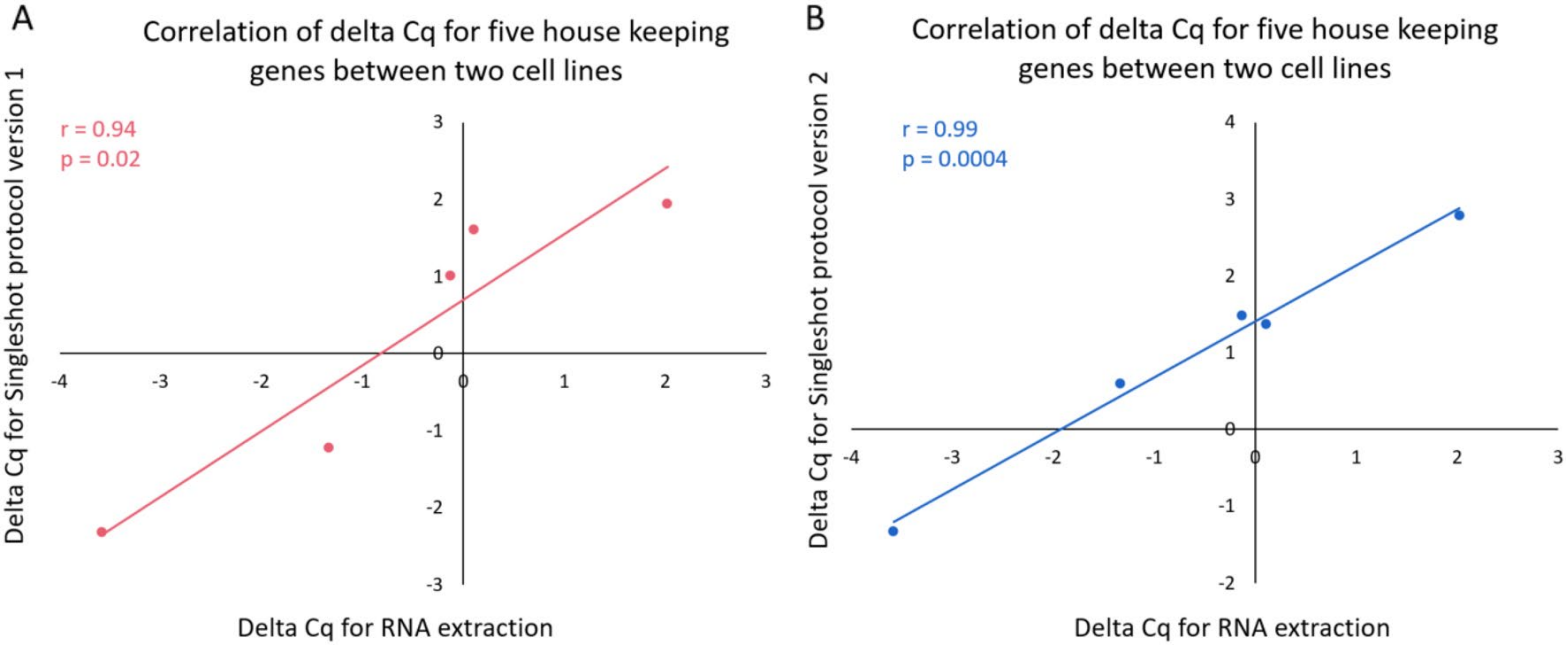
Correlation between delta Cq values (A549-BEAS-2B) from extracted RNA and lysate for both the version 1 and 2 lysis protocols in two different cell lines (BEAS-2B (**A**) and A549 (**B**)).

Next, we evaluated conservation of gene expression fold changes between both cell lines for all methods. For each method, we calculated delta Cq-values between cell lines for each of the five house keeping genes and compared these values between methods (Fig. 3). While we observed a significant correlation between fold changes obtained on extracted RNA and fold changes obtained on lysate for both lysis protocols, correlation was higher with the lysis protocol version 2 (v1: R = 0.94, *P* = 0.02; v2: R = 0.99, *P* = 0.0004), but this difference was not significant.

### Direct lysis of spheroids

**Figure 4:**
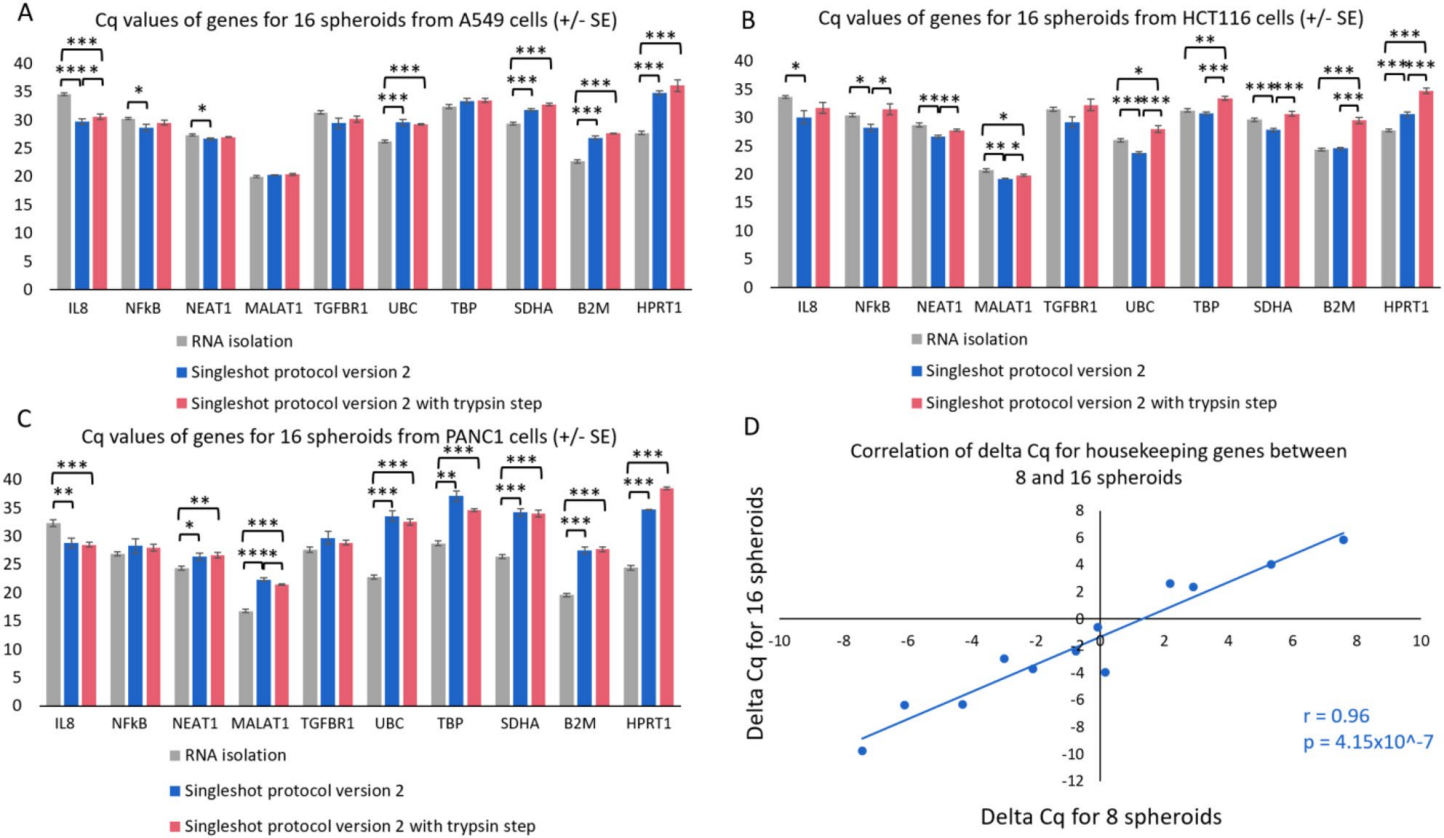
Cq values for 10 genes measured in three cell lines (A549 (**A**), HTC116 (**B**) and PANC1 (**C**)) on RNA extractions (grey) and on lysates generated using the Singleshot protocol version 2 (blue) or lysates generated using the Singleshot protocol version 2 and an additional Trypsin step (red). Data are presented as mean ± SE with *P < 0.05; **P < 0.01 and ***P < 0.001 (n = 3). Delta Cq values of house keeping genes between cell lines for 8 and 16 spheroids (**D**).

To assess performance of direct lysis on alternative 3D culture models, we applied lysis protocol version 2 on spheroids. Compared to the RWV-based 3D culture models, spheroids are dense cell clusters (supplemental Fig. 1B). Moreover, our sensitivity experiments with the 3D models suggested that sensitivity is compromised when performing direct lysis and that manipulations aimed at improving lysis (additional mixing step introduced in version 2 of the protocol) can, at least in some cell lines, improve sensitivity, we decided to also evaluate direct lysis with an upfront trypsin digestion to help break up the spheroids. Experiments were performed on spheroids from three different cell lines: A549, HCT116 and PANC1 and results were compared to standard RNA extraction. In contrast to the 3D cell cultures generated in a rotating wall vessel, direct lysis did not result in a consistent drop in sensitivity compared to standard RNA extraction. In A549 and PANC1 cells, direct lysis (protocol version 2 without trypsin) was less sensitive for 4/10 and 7/10 genes respectively, while in HCT116 cells, direct lysis was less sensitive for just 1/10 genes tested. For two cell lines (A549 and PANC1) there was no significant difference in Cq values with or without the upfront Trypsin step for 9/10 genes when lysing 16 spheroids (Fig. 4A and C). For HCT116 cells, Trypsin treatment unexpectedly resulted in a significant increase in Cq values for 8/10 genes (Fig. 4B, average delta Cq = 2.91). These trends were also witnessed when lysing 8 spheroids (supplemental Fig. 4). To further assess quantitative performance of the lysis procedure, we seeded 8 or 16 spheroids from each of the three cell lines, performed lysis and RT-qPCR for 4 reference genes, and evaluated preservation of fold changes between cell lines for the 8 and 16 spheroid conditions (Fig. 4D). Fold changes (calculated for each gene and each combination of 2 cell lines) were significantly correlated between the 8 and 16 spheroid conditions (R = 0.96, *P* = 4.15E − 07), confirming the quantitative performance of the lysis procedure on spheroid 3D cultures.

## Discussion

Three-dimensional cell cultures have gained increasing interest in various research fields to study biological processes and molecular functions, given their enhanced physiological relevance compared to conventional 2D monolayer cultures. Nevertheless, many common protocols in molecular biology need some degree of optimization to work efficiently on these types of cell culture. In this paper, we present an optimized protocol for direct lysis and RT-qPCR of rotating wall vessel derived 3D cell cultures. We demonstrated that a protocol commonly used on 2D cell cultures, SingleShot, shows strongly reduced sensitivity for qPCR-based gene expression quantification. A simple modification of the lysis step (by thorough mixing of lysis buffer and 3D cultures) substantially improved detection sensitivity for a subset of genes without impacting gene expression quantification accuracy. Most likely, the additional mixing step improves exposure of the cells in the 3D aggregates to the lysis reagent. We note that we also avoided carryover of microcarrier beads to the RT and qPCR reaction. As there are multiple types of 3D cell cultures, we also tested direct lysis (with and without Trypsin) on spheroids, another type of 3D cell culture. In contrast to what we expected, Trypsin treatment prior to lysis did not improve sensitivity and even proved a disadvantage for the HCT116 spheroids. Nevertheless, we could validate quantitative performance of the lysis protocol on spheroids by demonstrating that fold changes between samples are independent of the number of seeded spheroids.

While our results demonstrate the feasibility of direct lysis on different 3D culture models, model specific optimization of individual steps in the protocol may be required. One example is the input volume of lysate in the RT reaction. For 3D cultures derived from a rotating wall vessel, we observed lower Cq values (higher sensitivity) with 4 µl lysate input in RT compared to 8 µl input (data not shown), suggesting inhibition of RT when lysate input is too high. In contrast, lysates from spheroids did not appear to induce inhibition, as Cq-values were lower with 8 µl lysate input than with 4 µl lysate input. Other parameters, like cell density or lysis incubation time, may also impact performance and require further optimization on a per model basis. Although classic RNA extraction (gold standard) is more sensitive, direct cell lysis is an attractive time- and cost efficient alternative when processing dozens to hundreds of 3D cell culture samples, as labor intensive cell harvesting and RNA extraction are avoided. We demonstrate that direct lysis of 3D cell cultures is feasible and that minor modifications to the protocol can improve detection sensitivity for some genes and some cell lines.

## Methods

### Cell culture

3D lung epithelial cell cultures were generated using the rotating wall vessel (RWV) bioreactor system as described previously^27^. Briefly, A549 (adenocarcinoma alveolar lung epithelial cell line-ATCC CCL-185) were cultured in GTSF-2 medium (HyClone, Logan, UT) supplemented with 1.5 g/L sodium bicarbonate (Sigma-Aldrich), 10% fetal bovine serum (FBS, Life technologies), 2.5 mg/L insulin transferrin sodium selenite (Lonza) and 1% penicillin–streptomycin (Life Technologies), and BEAS-2B cells (normal human bronchial epithelium-ATCC CRL-9609) were grown in RPMI medium (Gibco, 52,400) supplemented with 1% penicillin–streptomycin. Both cell lines were grown in a 37 °C humidified incubator under 5% CO_2_. After 11 to 14 days of culturing in the RWV, 3D A549 and 3D BEAS-2B cells were transferred into 96-well plates at a concentration of 250,000 cells/well. To determine the cell concentration, cells were first washed three times with HBBS (to remove medium that could inhibit Trypsin) and detached with Trypsin (3 min incubation). 10 µl of cell suspension was combined with 10 µl of trypan blue and used to count cells with a Burker chamber. Singleshot lysis or conventional RNA extraction protocols were applied 24 h after seeding.

Spheroids were generated from three cell lines: A549 (cat. no. CCL-185), HCT116 (cat no. CCL-247) and PANC1 (cat. no. CRL-1469) all purchased from the American Type Culture Collection. All cells were cultured in DMEM (cat. no. 41965039, ThermoFisher) supplemented with 10% heat-inactivated fetal bovine serum (FBS) (cat. no. ATCC-30–2030, LGC Standards), 100 IU/ml penicillin and 100 mg/ml streptomycin (cat. no. 15070063, ThermoFisher). Cells were expanded and maintained as a monolayer at 37 °C in an atmosphere of 5% CO2 in air and passaged at 80% confluence. For spheroid formation, the U-shaped, 384-well ULA plates (cat. no. MS-9384UZ, S-bio) were seeded with a suspension of 80 µl cell culture media with 1 × 10^3^ cells per well (HCT116) or 2 × 10^3^ cells per well (A549 and PANC1). The culture media used was DMEM HG (25 mM glucose) (cat. no. 41965039, ThermoFisher), supplemented with 10% FBS, 100 IU/ml penicillin and 100 mg/ml streptomycin. The 384-well ULA plates were sealed with Breathe-Easy semipermeable tape (cat. no. BEM-1, Diversified Biotech) to prevent evaporation. The spheroids were cultured at 37 °C in an atmosphere of 5% CO2 under normoxia. Spheroids were lysed after seven days.

### Cell lysis

Direct cell lysis was performed using the SingleShot kit (BioRad) according to the manufacturer’s instructions (referred to as ‘protocol version 1’)^26^. Before removing the media or the PBS, 96 well plates were spun for 5 min at 2300 rpm. This was a step recommended by the manufacturer when working with non-adherent cells. To optimize the cell lysis protocol to improve lysis efficiency (referred to as the ‘protocol version 2’), 3D cultures were pipetted up and down vigorously when adding the lysis master mix, which was also suggested by the manufacturer for non-adherent cells. In addition, only 30 µl of lysate was transferred to a PCR plate to avoid carryover of the microcarrier beads.

For spheroid lysis we used 8 or 16 spheroids. Spheroids were collected in Eppendorf tubes, left to sink out and supernatant was removed. Spheroids were either trypsined or not, after that the same steps were followed as for the rotating wall vessel derived 3D cultures.

### RNA extraction

RNA extraction was performed using the microRNeasy kit from Qiagen according to the manufacturer’s instructions. A DNase treatment on column (RNAse-Free DNase set with DNase I from Qiagen, catalogue number = 79,254) was also performed.

### cDNA synthesis

cDNA synthesis was performed using the iScript Advanced cDNA Synthesis Kit from Bio-Rad according to the manufacturer’s instructions. Input for these cDNA synthesis reactions was either 500 ng for a 20 µl reaction for isolated RNA, or 4 µl of crude cell lysate from rotating wall vessel derived 3D cultures and 8 µl of crude cell lysate from the spheroids for a 20 µl reaction following the manufacturer’s recommendations. It may be valuable to optimize this input volume for every different cell type and density by testing input volumes between 4 and 9 µl as stated in the manual.

### qPCR

All qPCR reactions were performed in 384-well plates using the LightCycler 480 instrument (Roche). Reactions with cDNA derived from extracted RNA were performed in a total volume of 5 µl, comprising 2.5 µl 2 × SsoAdvancedUniversal SYBR Green Supermix (Bio-Rad), 2.5 ng/µl cDNA, and 0.25 µl of each forward and reverse primer (5 µM). For the reactions with lysates, 2 µl input was used of a fourfold dilution of the cDNA. A comparable amount of the lysate or RNA was used as input for the qPCR for the DNA contamination experiment. As reference genes the following primers were used: TBP (FWD: CACGAACCACGGCACTGATT;REV: TTTTCTTGCTGCCAGTCTGGAC), UBC (FWD: ATTTGGGTCGCGGTTCTTG ;REV:TGCCTTGACATTCTCGATGGT), SDHA (FWD:TGGGAACAAGAGGGCATCTG;REV:CCACCACTGCATCAAATTCATG), HPRT1 (FWD: TGACACTGGCAAAACAATGCA; REV: GGTCCTTTTCACCAGCAAGCT) and B2M (FWD: TGCTGTCTCCATGTTTGATGTATCT; REV: TCTCTGCTCCCCACCTCTAAGT). For the biologically relevant genes we used the following primers: IL8 (Bio-Rad primer qHsaCED0046633), NFkB (Bio-Rad primer qHsaCED0002379), NEAT1 (FWD: GGAGAGGGTTGGTTAGAGAT ; REV: CCTTCAACCTGCATTTCCTA), MALAT1 (FWD: AGTTCGTGGTGAAGATAGGA; REV: TAGCTTCCTTCACCAAATCG), and TGFBR1 (FWD: TCAGCTCTGGTTGGTGTCAG; REV: ATGTGAAGATGGGCAAGACC). For the DNA contamination experiment the following primers were used: NEUROD1 (FWD: CTTCTGCCGCCTGAAAGG; REV:CCTGGAACCACGTGACCTG), XRCC3(FWD:CTTGATTCTTTCTAGCCTTGG; REV:GGTTGACACTTTGATGGATAC), PLAT (FWD:GCGTGGCTTCTCTCTGATCC; REV:GAGCTCTGGCTTTTGCATCTG) and MTHFD2 (FWD: GTTCCCTTACTGGGTGGTGCTA; REV: AGTTACTGCTTCAACCACGTGATC). All primers have been wet lab validated for specificity and efficiency. The qPCR protocol in the LightCycler 480 consisted of enzyme activation (2 min at 95 °C), amplification (44 cycles of 5 s 95 °C, 30 s 60 °C and 1 s 72 °C), a melt curve cycle (5 s at 95 °C and 1 min at 60 °C) and cooling (3 min at 37 °C).

## Supplementary Information

Supplementary Information.

## Data Availability

The datasets generated during and/or analyzed during the current study are available from the corresponding author on request.
