# Supplementary material for: Direct lysis of 3D cell cultures for RT-qPCR gene expression quantification
Source: Sci Rep. 2023 Jan 27;13:1520. doi: 10.1038/s41598-023-28844-1 (PMC9883454; doi:10.1038/s41598-023-28844-1)
Supplement: Supplementary file 1 — Supplementary Information. [file 41598_2023_28844_MOESM1_ESM.pdf]

Supplemental figures

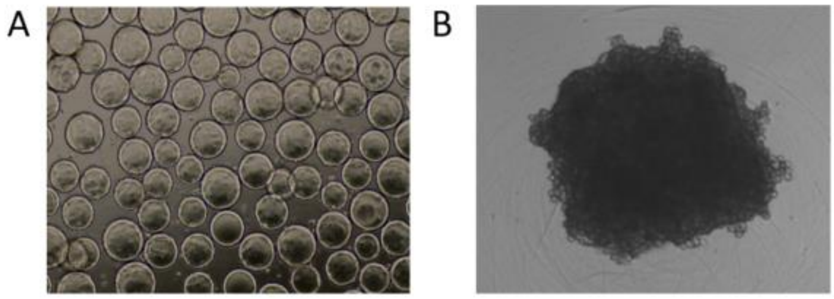

Supplemental figure 1. Microscopic picture of rotating wall vessel derived 3D BEAS-2B cultures (A) and spheroid from PANC1 cultures (B).

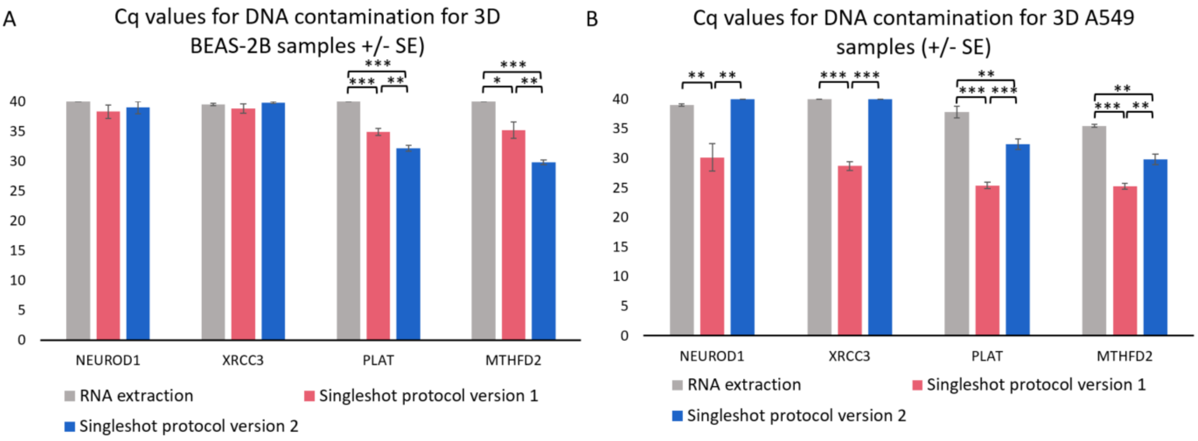

Supplemental figure 2. DNA contamination, represented by the Cq values of four genes, quantified on cell lysates or extracted RNA without including a reverse transcription step. Results are shown for both cell lines (BEAS-2B (A) and A549 (B)) on lysates generated with the version 1 (red) and version 2 (blue) lysis protocol and extracted RNA (grey) with p<0.05 = *; p<0.01 = ** and p<0.001 = ***.

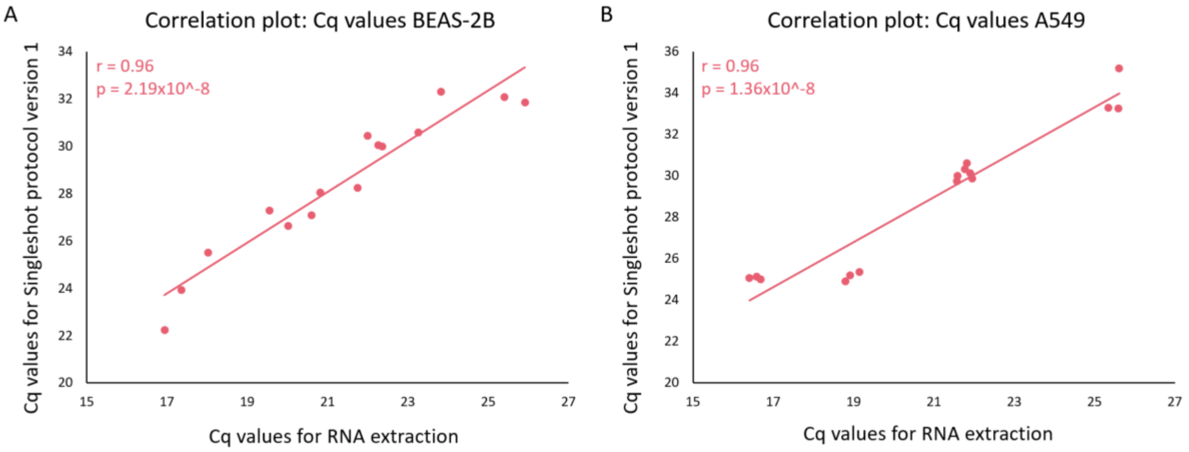

Supplemental figure 3. Correlation between Cq values for material derived from Singleshot lysis with protocol version 1 and from RNA extraction for two different cell lines (BEAS-2B (A) and A549 (B)).

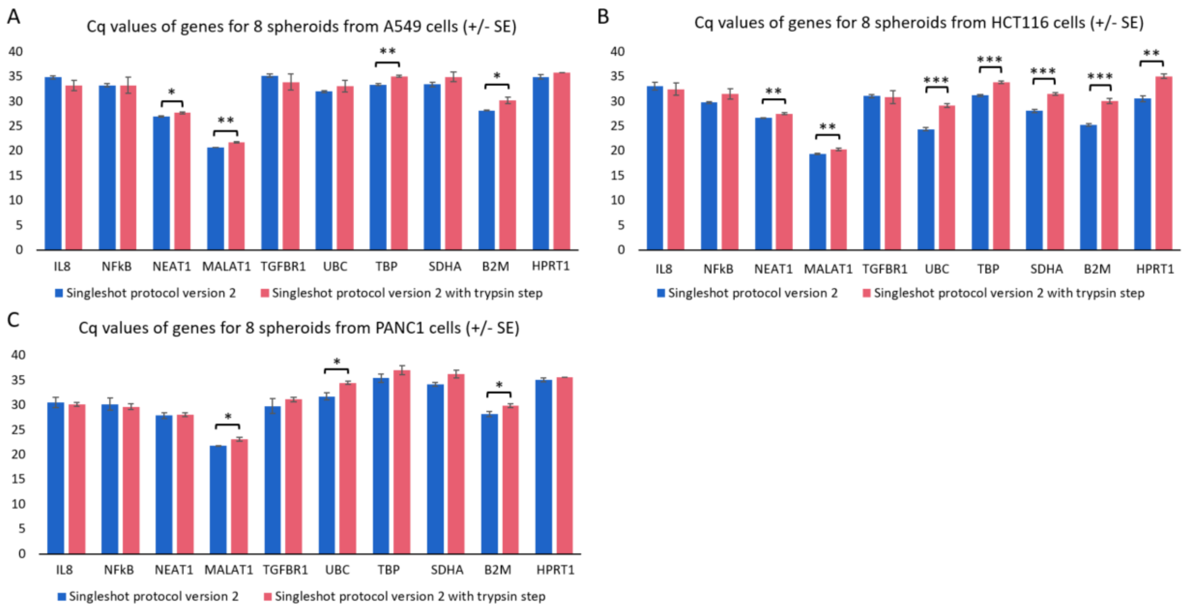

Supplemental figure 4. Cq values for 10 genes measured in three cell lines (A549 (A), HTC116 (B) and PANC1 (C)) on lysates generated using the Singleshot protocol version 2 (blue) or lysates generated using the Singleshot protocol version 2 and an additional Trypsin step (red). Data are presented as mean ± SE with $p<0.05$ = *; $p<0.01$ = ** and $p<0.001$ = ***(n=3).
